# Pharmacological inhibiting STAT5 for the treatment of FLT3‐ITD‐positive acute myeloid leukemia with triciribine phosphate monohydrate

**DOI:** 10.1002/mco2.294

**Published:** 2023-06-21

**Authors:** Hong Wu, Juan Ge, Fengming Zou, Zongru Jiang, Beilei Wang, Xinyu Yuan, Kaili Long, Jing Liu, Wenchao Wang, Qingsong Liu

**Affiliations:** ^1^ Anhui Province Key Laboratory of Medical Physics and Technology, Institute of Health and Medical Technology, Hefei Institutes of Physical Science Chinese Academy of Sciences Hefei Anhui China; ^2^ Hefei Cancer Hospital Chinese Academy of Sciences Hefei Anhui China; ^3^ University of Science and Technology of China Hefei Anhui China

Dear Editor,

Approximately 25% of acute myeloid leukemia (AML) patients carry FLT3‐ITD oncogenic mutations.^1^ Due to the essential role of the transcription factor STAT5 for the survival and proliferation of AML cells with this specific mutation, excessively active STAT5 can lead to drug resistance to FLT3 inhibitor.^2^, ^3^ Thus, STAT5 is a valuable target for combating FLT3‐ITD oncogenic mutations and overcoming drug resistance. Although several STAT5 inhibitors have been discovered, none of them have reached the clinical stage to treat FLT3‐ITD mutant AML, which is mainly due to their insufficient potency for further clinical transformation. Therefore, new potent STAT5 inhibitors are still in great need.

Using drug repositioning strategy, we discovered that triciribine phosphate monohydrate (TCN‐PM), the active metabolite of an AKT inhibitor triciribine with promising pharmacological efficacy in clinic trials, could specifically inhibit the growth of FLT3‐ITD mutant AML cancer cells as well as FLT3‐ITD primary AML cells (Figure 1A). Moreover, TCN‐PM significantly extended the animal survivals in FLT3‐ITD mutant MV‐4‐11 cell‐derived mouse bone marrow engraftment model (Figure 1B). Together, our study suggested that TCN‐PM could be a potent agent of FLT3‐ITD AML treatment.

**FIGURE 1:**
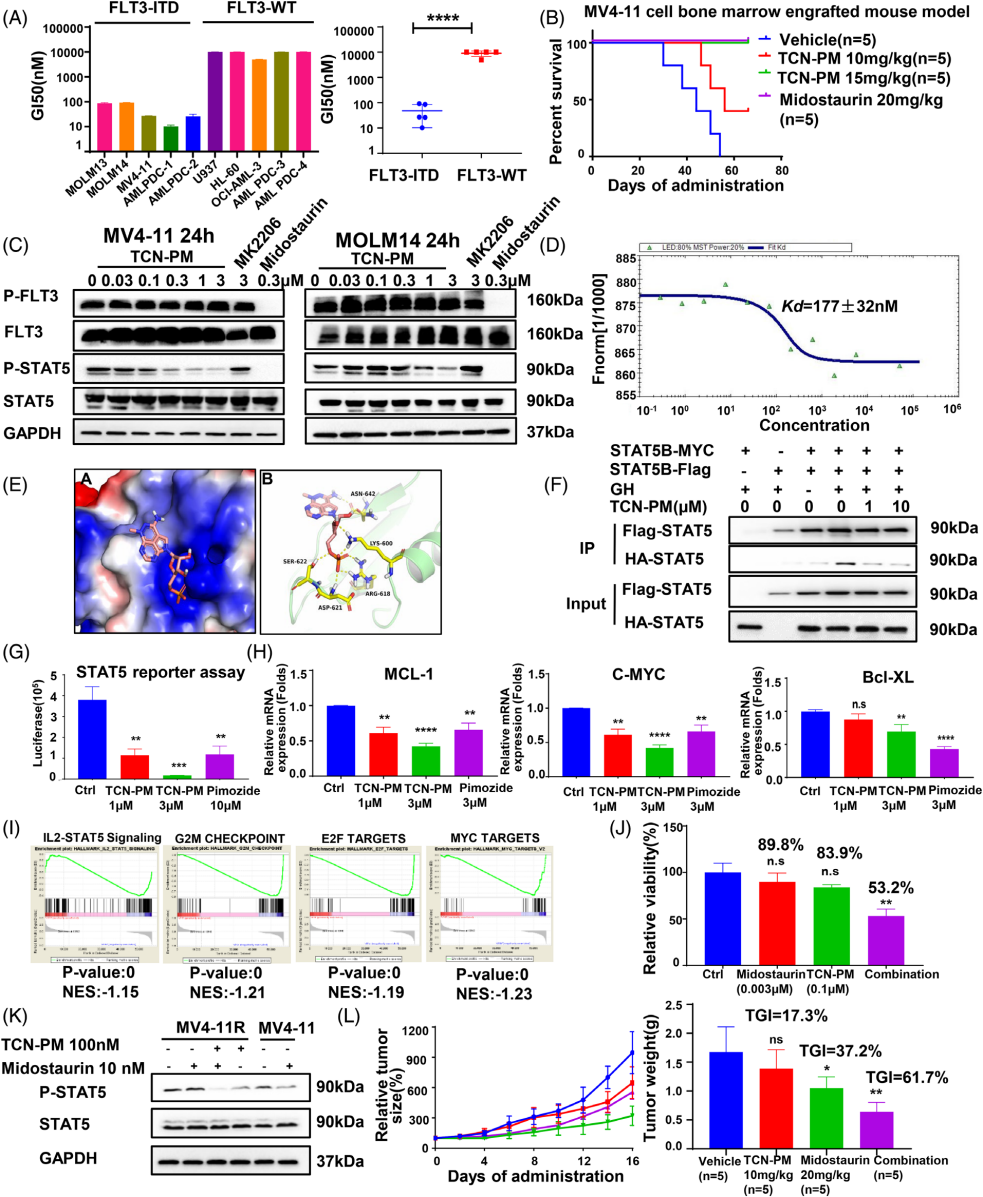
Characterization of TCN‐PM as a potent STAT5 inhibitor. (A) Antiproliferative effects of TCN‐PM against AML cancer cells and primary cells. (B) Antitumor efficacy of 10 mg/kg, 15 mg/kg TCN‐PM, and 20 mg/kg Midostaurin in MV4‐11 cell bone marrow engrafted mouse model. (C) Inhibitory effect of TCN‐PM on FLT3‐mediated signal pathway in FLT3‐ITD AML cells. (D) MST assay for the affinity between TCN‐PM and purified STAT5B protein. (E) Predicted binding mode of TCN‐PM to STAT5B (PDB: 6mbz) based upon molecular modeling. (F) TCN‐PM inhibited growth hormone (GH)‐induced dimerization of STAT5 in 293T cells. (G) Luciferase activity was determined in 293T cells transfected with reporter plasmids for STAT5. (H) mRNA expression of STAT5 target genes was analyzed by RT‐PCR. (I) Pathway analysis of gene on TCN‐PM treated MV4‐11 cells. (J) Antiproliferative effects of TCN, Midostaurin, or their combination on MV4‐11R cells. (K) Inhibitory effects of TCN‐PM, Midostaurin, and TCN‐PM in combination with Midostaurin on phosphorylation of STAT5 in MV4‐11 cells and MV4‐11R cells. (L) In vivo antitumor effects of 10 mg/kg TCN‐PM, 20 mg/kg Midostaurin, or their combination on MV4‐11R xenograft mouse model. Error bars mean ± SEM, *n* = 5. *p < 0.05; **p < 0.01; ***p < 0.001; ****p < 0.0001.

To investigate whether TCN‐PM exerts its inhibitory activity solely through AKT, we treated FLT3‐ITD and FLT3‐WT AML cells with another AKT inhibitor MK2206. However, we did not observe the same effect as that of TCN‐PM (Figure S1A). MK2206 did not selectively target FLT3‐ITD AML cells, while it was able to inhibit both FLT3‐WT and FLT3‐ITD AML cells. We also constructed a stable MOLM13 cell lineoverexpressing ATK‐T308D/S473D mutant, which has been reported to be resistant to TCN‐PM, and found that TCN‐PM could still suppress the cell proliferation even though it was unable to block AKT activation (Figures S1B and S1C). These results showed that TCN‐PM inhibits the proliferation of FLT3‐ITD cells by targeting an unknown target besides AKT.

To identify the direct target of TCN‐PM, we investigated the FLT3‐mediated signaling pathway and found that TCN‐PM could decrease STAT5 phosphorylation but had no effect on FLT3 autophosphorylation (Figure 1C), which indicated that TCN‐PM may be a STAT5 inhibitor. To verify that TCN‐PM displays its antiproliferation effect against FLT3‐ITD AML cells through STAT5, we then performed a microscale thermophoresis (MST) assay to measure the direct binding affinity between TCN‐PM and STAT5B, which is one of the two STAT5 proteins known to play important roles in the pathogenesis of hematological malignancies.^4^ The equilibrium dissociation constant (*K*_d_) value was determined to be 177 ± 32 nM (Figure 1D), suggesting that TCN‐PM has a high binding affinity with STAT5B. In support of this, the cellular thermal shift assay result confirmed that TCN‐PM robustly improved STAT5B protein thermal stability, which provided additional evidence for the direct binding between TCN‐PM and STAT5B (Figures S2A and S2B).

To better understand the binding mode of TCN‐PM with STAT5B, we then applied computer‐aided structural analysis by docking the molecule onto the homology model of the STAT5B SH2 domain (PDB: 6mbz) using AutoDock. The modeling result showed that the phosphoryl group of TCN‐PM engaged in electrostatic interactions with the positively charged side chain residues Lys600 and Arg618 in a similar manner as ATP.^5^ In addition, one hydroxy of the phosphoryl group formed a hydrogen bond with Asp621, while the NH_2_ of the core moiety and the hydroxy group of the tetrahydrofuran ring formed two hydrogen bonds with Asn642 (Figure 1E). Consistent with the binding mode proposed by our docking analysis, the activities of TCN‐PM against the STAT5B Lys600Ala, Arg618Ala, and Asn642Ala mutants were markedly reduced compared with STAT5B wild‐type (Figures S2C and S2D). Additionally, when MV4‐11 cells were stimulated with IFNγ and IL‐6 to activate STAT1 and STAT3, TCN‐PM had no effect on either of their activities (Figure S2E). The above studies demonstrated that TCN‐PM can bind to STAT5 and specifically suppress STAT5 activity.

The mechanism by which TCN‐PM modulates STAT5 was then explored. We found that TCN‐PM substantially inhibited STAT5B dimerization induced by growth hormone (Figure 1F), and we observed dose‐dependent reduction of STAT5‐dependent transcription after TCN‐PM treatment using STAT5 promoter reporter assay (Figure 1G). Moreover, TCN‐PM reduced the expression levels of genes regulated by STAT5 such as MYC, Bcl‐xL, and Mcl‐1 at both mRNA and protein levels (Figures 1H and S3A). To verify our findings at the genomic level, we performed RNA sequencing and analyzed gene expression profiles using gene‐set enrichment analysis to further investigate the genetic mechanism involved in TCN‐PM treatment. Consistently, the results showed that the IL‐2‐STAT5 signaling pathway was downregulated following TCN‐PM treatment (Figure 1I). Furthermore, TCN‐PM treatment decreased the expression of E2F targets, G2M checkpoint targets, and MYC targets (Figure 1I), implying that STAT5 inhibition can induce cell‐cycle arrest and apoptosis. The cell cycle and biochemical experiments also showed that TCN‐PM could induce cell cycle arrest in G0–G1 phase and trigger apoptosis through caspase‐3 activation and PARP cleavage in both MOLM14 and MV4‐11 cells (Figures S3B and S3C). The results collectively demonstrated that TCN‐PM inhibits STAT5 activation by preventing its dimerization and transcription regulation.

There is increasing evidence that STAT5 overactivation contributes to FLT3 inhibitor resistance, and we also found that the level of phosphorylated STAT5 was increased in Midostaurin resistant MV4‐11 cells (MV4‐11R) (Figures S4A and S4B). To determine whether TCN‐PM can enhance the anticancer activity of Midostaurin to overcome drug resistance, we combined TCN‐PM with Midostaurin and found that the two drugs had a synergistic inhibitory effect on the growth of MV4‐11R cells (CI index = 0.06). TCN‐PM enhanced the antiproliferation efficacy of Midostaurin (relative viability Midostaurin + TCN: 53.2% vs. Midostaurin: 89.8%) (Figure 1J). Besides, TCN‐PM combined with Midostaurin could help decrease STAT5 activity more efficiently than Midostaurin alone (Figure 1K). Subsequently, we used a MV4‐11R xenograft mouse model to evaluate the effect of TCN‐PM in combination with Midostaurin on tumor growth in vivo. It was found that the combination significantly increased the tumor growth inhibition (TGI) value to 61.7% compared with TCN‐PM (TGI = 17.3%) or Midostaurin (TGI = 37.2%) treatment alone without affecting body weight (Figures 1L and S4C). Overall, these results showed that TCN‐PM potently enhances the antitumor effect of Midostaurin to overcome drug resistance.

In this study, we showed that FLT3‐ITD AML cells can be selectively inhibited by TCN‐PM, which exhibits a promising inhibition efficacy against FLT3‐ITD AML both in vitro and in vivo. The mechanistic research has shown that the effect of TCN‐PM in FLT3‐ITD AML cells is achieved by direct targeting of STAT5. Those findings add more convincing evidence on the importance of STAT5 as a promising target for treatment of FLT3‐ITD AML and overcoming drug resistance. Besides, TCN‐PM has been clinically investigated against advanced hematologic malignancies and showed acceptable safety profile in patients. Although the data from phase I/II trials of TCN‐PM therapy in advanced hematologic malignancies patients are encouraging, the clinical advance of TCN‐PM is limited due to the variable AKT activities in AML patients and the lack of biomarker for drug sensitivity. Our findings not only identified TCN‐PM as a potent STAT5 inhibitor, but also provided evidence for extending the use of this drug to AML patients carrying FLT3‐ITD mutation, which could serve as a biomarker for more precise application of TCN‐PM in clinical therapy.

## AUTHOR CONTRIBUTIONS

H. W., and J. G. performed the molecular biology and biochemical experiments; F. Z. and Z. J. performed the animal study; B. W. performed the modeling study; X. Y. and K. L. performed the antiproliferation tests; H. W. drafted the manuscript; W. W., J. L., and Q. L. supervised the project and revised the manuscript. All authors reviewed the paper. All authors have read and approved the final manuscript.

## ETHICS STATEMENT

The Animal Ethics Committee of Hefei Institutes of Physical Science, Chinese Academy of Sciences approved the study (number: DWLL‐2020‐34). The Medical Ethics Committee of CAS Hefei Cancer Hospital approved the study (number: PJ‐KY2022‐005).

## FUNDING INFORMATION

This work was supported by the National Natural Science Foundation of China (Grant Nos. 82104239 and 32171479), the Natural Science Foundation of Anhui Province (Grant No. 2108085QH377), and the CASHIPS Director's Fund (Grant Nos. YZJJZX202011 and YZJJ2021QN38). We are also grateful for the support of the Youth Innovation Promotion Association of CAS support (No. 2019437) for H. W.

## CONFLICT OF INTEREST STATEMENT

All authors declare no conflict of interest.

## Supporting information

Supplementary information

## Data Availability

The data are available from the corresponding author upon reasonable request.
