# Supplementary material for: Pharmacological inhibiting STAT5 for the treatment of FLT3‐ITD‐positive acute myeloid leukemia with triciribine phosphate monohydrate
Source: MedComm (2020). 2023 Jun 21;4(4):e294. doi: 10.1002/mco2.294 (PMC10285034; doi:10.1002/mco2.294)
Supplement: Supplementary file 1 — Supplementary information [file MCO2-4-e294-s001.docx]

SUPPLEMENTARY MATERIALS

**Enhancing therapeutic efficacy of Triciribine phosphate monohydrate through targeting STAT5 in FLT3-ITD AML**

Hong Wu^1,3#^, Juan Ge^1,2#^, Fengming Zou^1,3#^, Zongru Jiang^1^, Beilei Wang^1,3^, Xinyu Yuan^1,2^, Kaili Long^1,2^, Jing Liu^1,2,3^*, Wenchao Wang^1,2,3^*, Qingsong Liu^1,2,3,4^*

1_._Anhui Province Key Laboratory of Medical Physics and Technology, Institute of Health and Medical Technology, Hefei Institutes of Physical Science, Chinese Academy of Sciences, Hefei, Anhui 230031, P. R. China

2.University of Science and Technology of China, Hefei, Anhui 230026, P. R. China

3. Hefei Cancer Hospital, Chinese Academy of Sciences, Hefei, Anhui 230031, P. R. China

Hong Wu, Juan Ge and Fengming Zou contributed equally to this work

*Corresponding authors. Institute of Health and Medical Technology, Hefei Institutes of Physical Science, Chinese Academy of Sciences, Hefei, Anhui 230031, P. R. China. E-mail address：[qsliu97@hmfl.ac.cn (Q.Liu)](mailto:qsliu97@hmfl.ac.cn(Q.Liu))

*Corresponding authors. Institute of Health and Medical Technology, Hefei Institutes of Physical Science, Chinese Academy of Sciences, Hefei, Anhui 230031, P. R. China. E-mail address：[wwcbox@hmfl.ac.cn](mailto:wwcbox@hmfl.ac.cn) (W.Wang)

*Corresponding authors. Institute of Health and Medical Technology, Hefei Institutes of Physical Science, Chinese Academy of Sciences, Hefei, Anhui 230031, P. R. China. E-mail address：[jingliu@hmfl.ac.cn (J.Liu)](mailto:jingliu@hmfl.ac.cn(J.Liu))

**This PDF file includes:**

Materials and Methods

Figures. S1 to S4

**Materials and Methods**

**Cell Lines and Cell culture:**

The human AML cancer cell lines MV4-11, MOLM13, and MOLM14 were provided by Dr. Scott Armstrong, Dana-Farber Cancer Institute (DFCI), Boston, MA, USA. U937, OCI-AML-3, and HL-60 cells were purchased from Cobioer Biosciences Co., Ltd. (Nanjing, China). MV4-11R were isolated by passaging the cells in growth medium containing increasing concentrations of Midostaurin. MV-4-11 and MV4-11Rcells were cultured in Iscove’s Modified Dulbecco’s Medium containing 10% FBS and 1% penicillin/streptomycin. MOLM13, MOLM14, U937, and HL-60 cells were cultured in RPMI1640 medium with 10% FBS and 1% penicillin/streptomycin. OCI-AML-3 cells were cultured with α-MEM media (Corning, USA) with 10% FBS and 1% penicillin/streptomycin. Human primary patient samples were obtained under approval of the First Hospital of Anhui Medical University, Anhui, China. All studies performed with human specimens were performed with approval from the CAS Hefei Cancer Hospital. Ethical approval and informed consent were obtained for the use of human samples. Primary AML cells were cultured in human acute myeloid leukemia cell medium (PRS-AMLM) (Hefei PreceDo pharmaceuticals Co. Ltd., Anhui, China).

**Antibodies and reagents:**

FLT3 antibody (#3462), Phospho-FLT3 (Tyr589/591) antibody (#60413), Phospho-Stat5 (Tyr694) antibody (#4322), Stat5 antibody (#25656)，c-Myc antibody (#5605), Bcl-xL antibody (#2764), PARP antibody(#9532), Mcl-1 antibody(#94296) and Caspase-3 antibody (#9665), Phospho-Akt (Ser473) antibody (#4060), Phospho-Stat1 (Ser727) antibody(#9177), Stat1antibody(#14994), Phospho-Stat3 (Tyr705) antibody(#9145), Stat3 antibody(#12640) were purchased from Cell Signaling Technology (Danvers, MA, USA).

**Proliferation and drug combination studies:**

5 × 10^3^ cells were plated in 96 well plate and treated with different compounds at the indicated concentration for 72 h. Cell viability was measured using Cell Titer–Glo assay (Promega, USA) according to the manufacturer’s instructions and the luminescence signals were then detected by fluorescence microplate reader (Perkin Elmer Envision).

**Quantitative real-time polymerase chain reaction (qPCR)**

After TCN-PM treatment as described, cells were collected, then mRNA was extracted using the RNeasy Mini Kit (Qiagen) and converted to cDNA using SuperScript III reverse transcriptase (Thermo Fisher). Real-time qPCR was carried out in a 96-well plate using SYBR green probes and a 7500 FAST Real-Time PCR system (Thermo Fisher). Relative gene expression was calculated by comparison to a GAPDH reference probe. PCR Primer used for qPCR:

C-MYC: Forward Primer GGCTCCTGGCAAAAGGTCA

Reverse Primer CTGCGTAGTTGTGCTGATGT

BCL-XL: Forward Primer GAGCTGGTGGTTGACTTTCTC

Reverse Primer TCCATCTCCGATTCAGTCCCT

MCL-1: Forward Primer TGCTTCGGAAACTGGACATCA

Reverse Primer TAGCCACAAAGGCACCAAAAG

**GSEA analysis**

MV4-11 cells were treated with 1 μM TCN-PM for 24 h and harvested for mRNA expression profiling by Novogene (Beijing, China). Differential expression analysis was performed using the DESeq2 R package (v1.23.0) and genes with an adjusted P-value <0.05 and absolute value of fold change >2 were assigned as differentially expressed. GSEA v3.0 and dataset were obtained from the Gene Set Enrichment Analysis website (http://software. broadinstitute.org/gsea/index.jsp).

**Cell cycle measurements**

MOLM14 and MV4-11 cells were treated with TCN-PM at indicated concentration for 24 h. Cells were harvested and fixed in 70% ethanol and stained with PI/RNase staining buffer (BD Pharmingen). Flow cytometry was performed using a FACS Calibur (BD), and the results were analyzed by ModFit software.

**Signaling pathway and apoptosis measurements**

MOLM14 and MV4-11 cells were treated with TCN-PM at indicated concentration for 12 h-48 h. Cells were harvested and lysed by RIPA (Beyotime) supplemented with protease/phosphatase Inhibitor Cocktail (Cell Signaling Technology). The suspension of cell lysis was boiled with 5 × loading buffer, proteins were load on SDS–PAGE and western blot using specific antibody.

**CETSA**

TCN-PM and DMSO were added to MV4-11 cell lysates and incubated for 1 h. Samples were then divided and transferred to PCR tubes for incubation at different temperatures for 3 min. Insoluble proteins were separated by centrifugation, and the soluble proteins were analyzed using Western blotting.

**MST**

The *Kd* value was measured using the Monolith NT.115 instrument (Nano Temper Technologies). TCN-PM at indicated concentration were incubated with 200 nM of purified STAT5B protein for 30 min in reaction buffer (50 mM HEPES, 10 mM MgCl2, 100 mM NaCl, pH 7.5, and 0.05% Tween 20). The samples were loaded into the Nano Temper glass capillaries, and MST was performed using 100% LED power and 80% MST power. The *Kd* value was calculated using the mass action equation via the Nano Temper software from duplicate reads of an experiment.

**Luciferase reporter gene assay**

STAT5 luciferase reporter GPL4 plasmid and pcDNA3.1 STAT5B plasmid were co-transfected into HEK293T cells. After 12 h, cells were plated in 96 well plate and treated with different compounds at the indicated concentration for 24 h, followed by measuring firefly luciferase activities using Bright-Glo reagent (Promega) with a plate reader ENVISION (MD).

**Determination of STAT5 dimerization by co-immunoprecipitation**

PcDNA4.1 STAT5B-Flag and pcDNA5.1 STAT5B-HA were co-transfected into HEK293T cells. After 12 h, cells were plated in 6 well plate and starved for 6 h, pretreated with TCN-PM at indicated concentrations or DMSO for 6 h, and stimulated with 500 ng/ml hGH for 20 min. Cells were collected and FLAG-tagged proteins were immunoprecipitated with anti-FLAG M2 Affinity Gel. The beads were washed at least 3 times with washing buffers and eluted with 2×loading buffer for western blot using specific antibody.

**Invitro-kinase assay**

pcDNA3.1 FLT3-ITD and pcDNA3.1 STAT5B were co-transfected into HEK293T cells. After 24 h, cells were plated in six well plate and treated with TCN-PM at indicated concentrations or DMSO for 24 h. Cells were collected and western blot using specific antibody.

**MV4-11 cells bone marrow engrafted mouse model**

Five-week-old female NOD-SCID mice were purchased from the GemPharmatech Co., Ltd. (Nanjing, China). All animals were housed in a specific pathogen free facility and used according to the animal care regulations of Hefei Institutes of Physical Science, Chinese Academy of Sciences (Hefei, China). NOD-SCID mice were intraperitoneal injection (i.p.) cyclophosphamide (CTX) 50 mg/kg daily for two days. Six million MV-4-11 cells in 0.2 mL IDMEM medium were injected through intravenous after 24 h. 10mg/kg ,15mg/kg TCN-PM were administered daily by i.p., and 20mg/kg Midostaurin were administered daily by peros (p.o.). Mice were monitored daily and were euthanized when moribund or at early signs of hind limb paralysis.

**MV4-11 cell subcutaneous Xenograft tumor model**

Four-week-old female Balb/c nude mice were purchased from GemPharmatech Co., Ltd (Nanjing, China). All animals were housed in a specific pathogen-free facility and used according to the animal care regulations of Hefei Institutes of Physical Science, Chinese Academy of Sciences (Hefei, China). The serial number for animal experiments in Ethic approval is DWLL-2020-34. Prior to implantation, cells were harvested during exponential growth. 1×10^7^ MV4-11 cells in IDMEM medium were formulated as a 1:1 mixture with Matrigel (BD Biosciences) and injected into the subcutaneous space on the right flank of Bal b/c nude mice. Daily oral administration was initiated when MV4-11 tumors had reached a size of 100-150 mm^3^. Animals were then randomized into treatment groups of 5 mice each for efficacy studies. Compound was delivered daily in a HKI solution (0.5% methocellulose/0.4% Tween80 in ddH_2_O). TCN-PM(10mg/kg) were administered daily by i.p., and Midostaurin(20mg/kg) were administered daily by p.o. Body weight and tumor growth were measured twice a day. Tumor volume was calculated as follows: tumor volume (mm^3^) = [(W^2^×L)/2] in which width (W) is defined as the smaller of the two measurements and length (L) is defined as the larger of the two measurements. Tumor volume on the day of drug administration is set as 100% for analysis.

**Statistical analysis**

All results are presented as the means ± SEM and Student’s t test was used for statistical analysis among the different groups; IC_50_ and GI_50_ values were calculated using Prism 8.0 (Graph Pad Software, San Diego, CA, USA) using the normalized dose response curve for inhibition (variable slope).


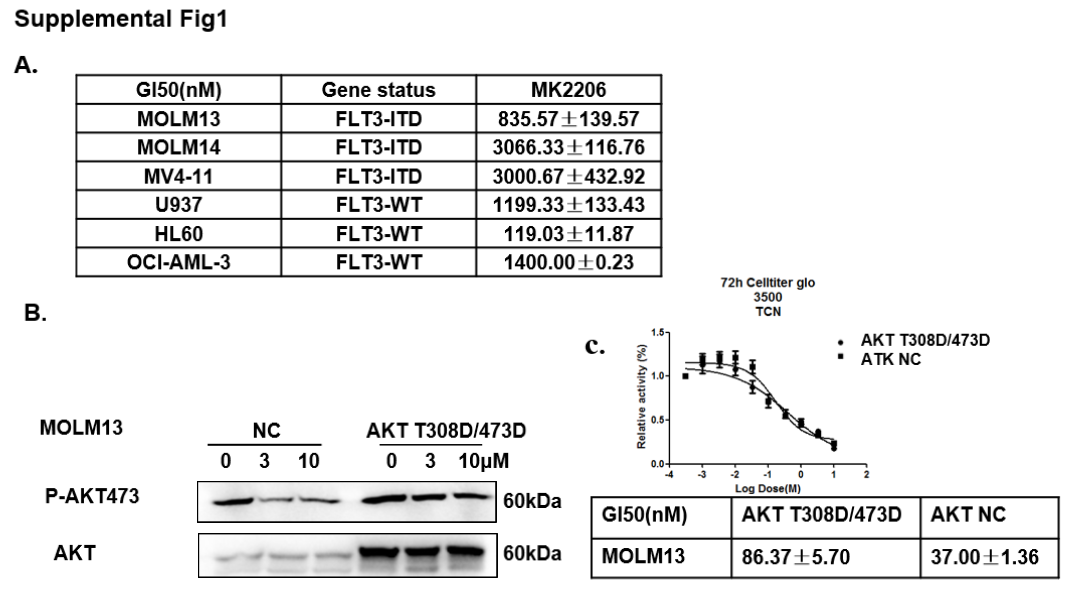


**Supplemental Fig1: TCN-PM exerts its inhibitory activity not through targeting AKT.**

A. Antiproliferative effects of MK2206 against AML cancer cells and primary cells. B. Inhibitory effects of TCN-PM on phosphorylation of ATK in MOLM13 and AKT T308/473D MOLM13 cells. C. Antiproliferative effects of TCN-PM against MOLM13 and AKT T308/473D overexpressed MOLM13 cells


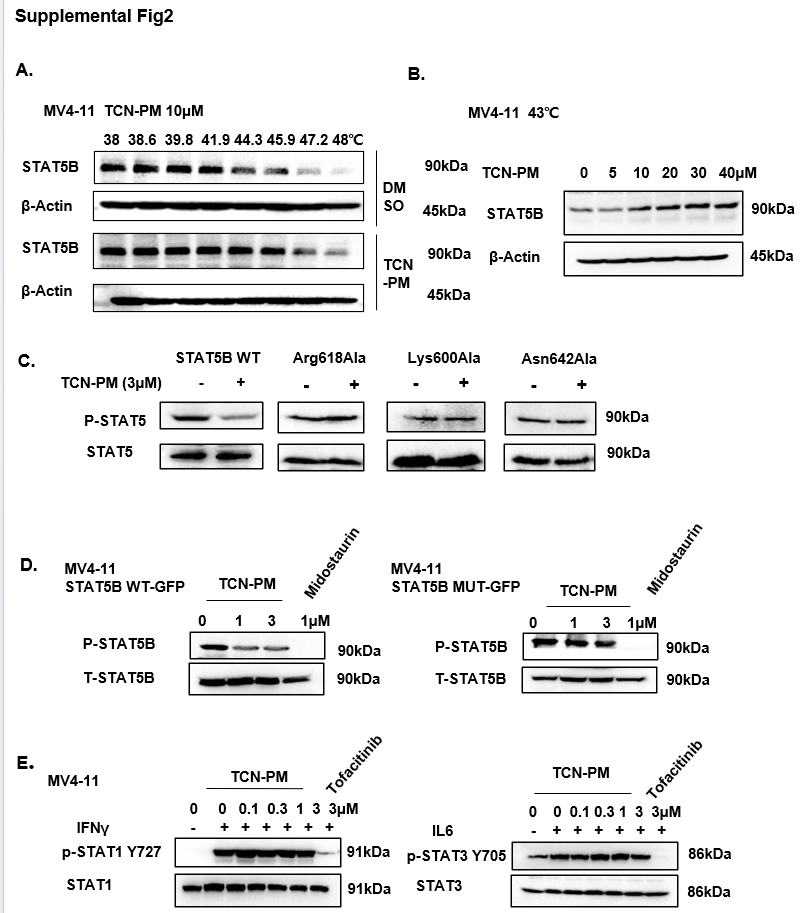


**Supplemental Fig2: Characterization of TCN-PM as a potent STAT5 inhibitor.**

A. The effect of TCN-PM on the stability of the STAT5B protein in a temperature-dependent manner was investigated using MV4-11cell lysate. B. The effect of TCN-PM on the stability of the STAT5B protein in a dose-dependent manner was investigated using MV4-11 cell lysate. C. Inhibitory effects of TCN-PM on phosphorylation of wild-type and mutant STAT5 proteins in 293T cells transfected with FLT3-ITD and wild-type or mutant STAT5 proteins（Lys600Ala,Arg618Ala,Asn 642Ala triple mutation）. D. Inhibitory effect of TCN-PM on phosphorylation of wild-type and mutant STAT5 proteins in MV4-11 cells transfected with STAT5B wt-GFP and STAT5B mutant-GFP overexpression virus. E. Inhibitory effect of TCN-PM on phosphorylation of STAT1 and STAT3 in MV4-11 cells stimulated with IFNγ and IL-6.


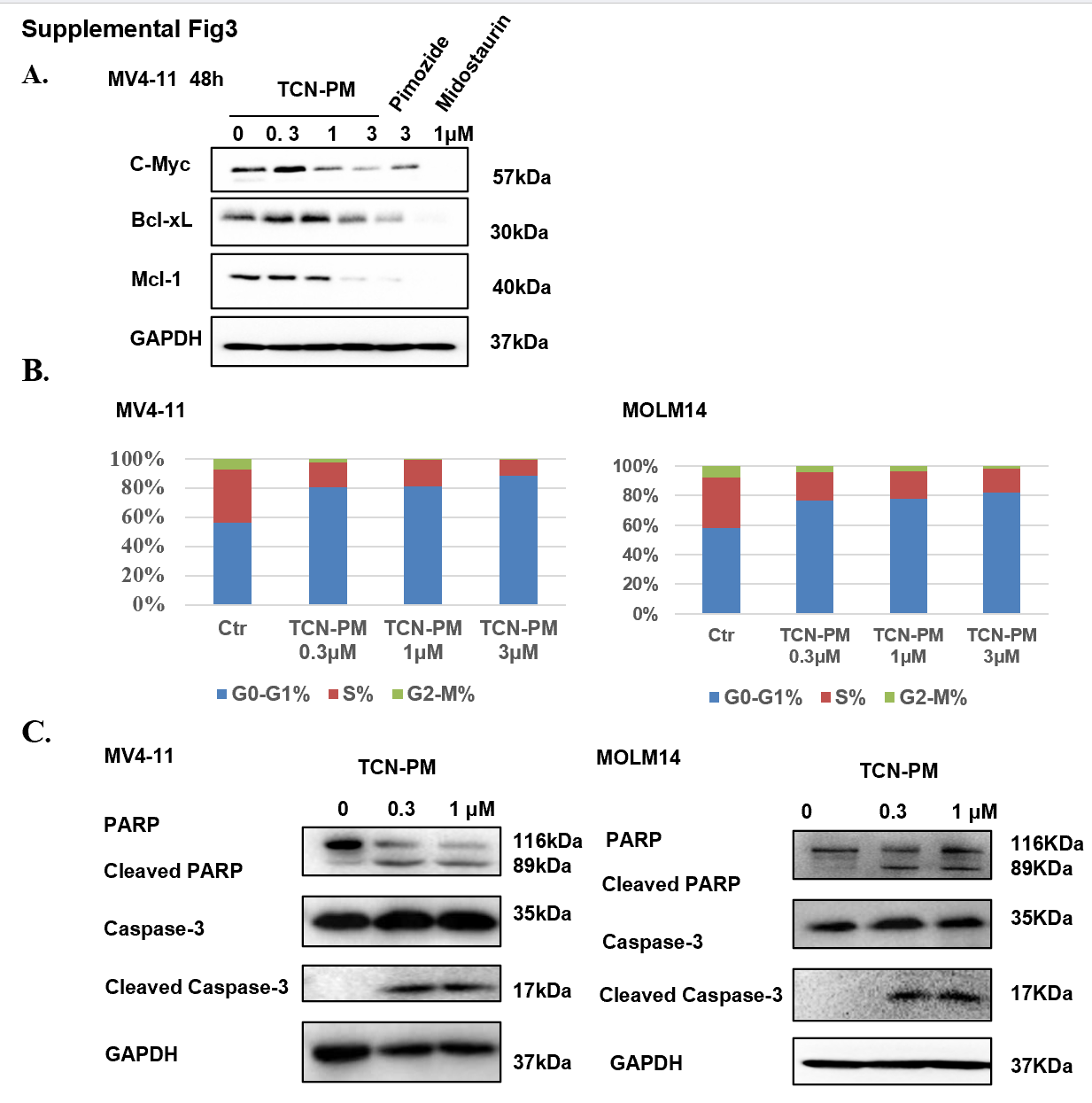


**Supplemental Fig3: TCN-PM induces cell cycle arrest and subsequent apoptosis.**

A. Protein expression of STAT5 target genes was analyzed by western blot. B. Cell cycle analysis of MV4-11 and MOLM14 cells treated with TCN-PM for 24 h. C. Western blot analysis for the expression of PARP, cleaved PARP, caspase-3, and cleaved caspase-3 in MOLM-14 and MV4-11 cells treated with TCN-PM for 24 h or 48 h.


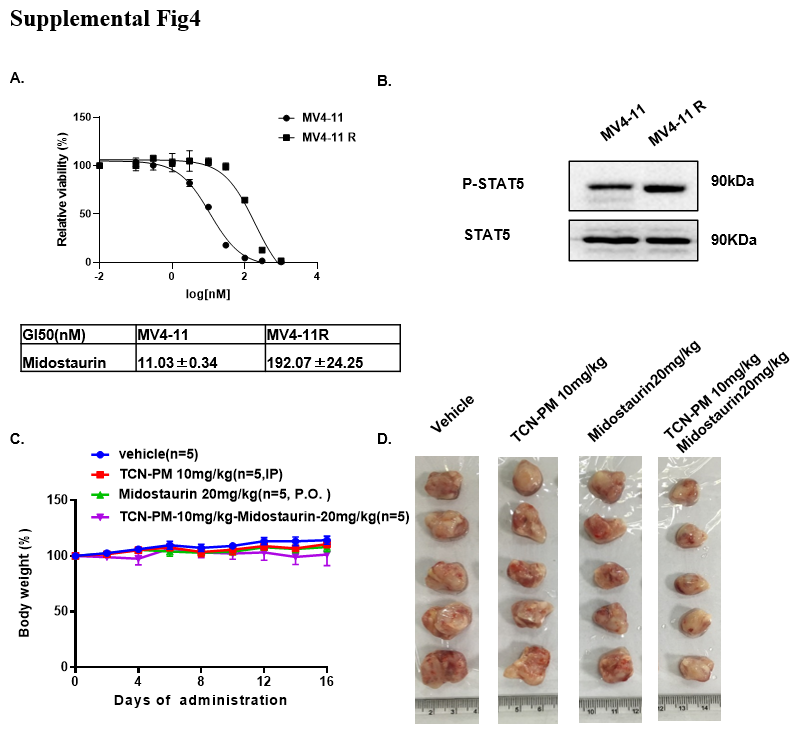


**Supplemental Fig4:** **Impact of TCN-PM on the growth of MV4-11R cells in vitro and in vivo.**

A. Antiproliferative effects of Midostaurin against MV4-11 and MV4-11R cells. B. Western blot analysis for the phosphorylation of STAT5 in MV4-11 and MV4-11R cells. C.Body weight measurement in each group after TCN-PM, Midostaurin or their combination treatment in MV4-11R xenograft mouse model. D. Tumor image of drug treatment in MV4-11R xenograft mouse model.
